# A field strength independent MR radiomics model to predict pathological complete response in locally advanced rectal cancer

**DOI:** 10.1007/s11547-020-01266-z

**Published:** 2020-08-24

**Authors:** Davide Cusumano, Gert Meijer, Jacopo Lenkowicz, Giuditta Chiloiro, Luca Boldrini, Carlotta Masciocchi, Nicola Dinapoli, Roberto Gatta, Calogero Casà, Andrea Damiani, Brunella Barbaro, Maria Antonietta Gambacorta, Luigi Azario, Marco De Spirito, Martijn Intven, Vincenzo Valentini

**Affiliations:** 1 Fondazione Policlinico Universitario “Agostino Gemelli” IRCCS, Rome, Italy; 2 Department of Radiation Oncology, University Medical Center Utrecht, Utrecht, The Netherlands; 3 Istituto di Radiologia, Università Cattolica del Sacro Cuore, Largo Francesco Vito 1, 00168 Rome, Italy

**Keywords:** Radiomics, Magnetic resonance imaging, Inter-scanner variability, Magnetic field intensity, Rectal cancer

## Abstract

**Purpose:**

Aim of this study was to develop a generalised radiomics model for predicting pathological complete response after neoadjuvant chemo-radiotherapy in locally advanced rectal cancer patients using pre-CRT T2-weighted images acquired at a 1.5 T and a 3 T scanner.

**Methods:**

In two institutions, 195 patients were scanned: 136 patients were scanned on a 1.5 T MR scanner, 59 patients on a 3 T MR scanner. Gross tumour volumes were delineated on the MR images and 496 radiomic features were extracted, applying the intensity-based (IB) filter. Features were standardised with *Z*-score normalisation and an initial feature selection was carried out using Wilcoxon–Mann–Whitney test: The most significant features at 1.5 T and 3 T were selected as main features. Several logistic regression models combining the main features with a third one selected by those resulting significant were elaborated and evaluated in terms of area under curve (AUC). A tenfold cross-validation was repeated 300 times to evaluate the model robustness.

**Results:**

Three features were selected: maximum fractal dimension with IB = 0–50, energy and grey-level non-uniformity calculated on the run-length matrix with IB = 0–50. The AUC of the model applied to the whole dataset after cross-validation was 0.72, while values of 0.70 and 0.83 were obtained when 1.5 T and 3 T patients were considered, respectively.

**Conclusions:**

The model elaborated showed good performance, even when data from patients scanned on 1.5 T and 3 T were merged. This shows that magnetic field intensity variability can be overcome by means of selecting appropriate image features.

## Introduction

Rectal cancer accounts for one third of colorectal cancers and is to date one of the leading causes of cancer death in the western world [1, 2].

Neoadjuvant chemo-radiotherapy (nCRT) followed by total mesorectal excision (TME) represents the standard of care for patients affected by locally advanced rectal cancer (LARC), defined as stage II (T3 or T4, node-negative, M0) and stage III (T3 or T4, node-positive, M0) rectal cancer.

In particular, nCRT reduces the risk of local recurrence and downsizes the primary tumour facilitating the subsequent successful surgical resection or allowing sphincter-preserving approaches. In a variable fraction of cases (8–30%), the downsizing effect is complete and a pathological complete response (pCR) after nCRT is reached [3–5].

The response after nCRT and outcomes are strongly correlated, as assessed in several pooled analyses, where complete responders show a better prognosis in terms of local control and disease free survival, compared to non-responders [6–8].

New therapeutic approaches, more conservative respect to the TME surgery, have currently under investigation for patients showed pCR after nCRT, such as local excision (LE) or watch and wait (W&W) [9–11].

To increase the number of locally advanced rectal cancer (LARC) patients with organ sparing treatment approaches, there is a growing interest in realising predictive models able to identify patients who will completely respond to nCRT before the start of therapy. These predictive models can be based on the analysis of clinical parameters, DNA sequences or radiomic parameters extracted by diagnostic images [12, 13].

An increasing number of studies focused on the possibility to predict pCR analysing magnetic resonance imaging (MRI), as this modality is generally the gold standard diagnostic imaging technique for rectal cancer [14, 15].

Different radiomics experiences performed on staging pre nCRT MRI have demonstrated that it is possible to predict complete response to treatment or early disease progression, relapse or distant progression in the first 3 months after radical surgery [16–19].

However, the application of radiomics to MRI is performed less often compared to radiomics on CT or positron emission tomography, due to the high complexity of the MRI signal processing, that require dedicated filtering and signal intensity standardisation procedures [20–22].

Another factor that obstructs the broad implementation of radiomics in daily clinical practice is the lack of evidence for the general applicability of the models for different MR scanner vendors and different magnetic field strengths [23].

Most of the imaging-based predictive models to date available are indeed strongly linked to the technical aspects of the used MR scanner [23, 24]. Aim of this study is to overcome these sources of variability, developing a radiomics model able to predict pCR in LARC patients analysing pre-CRT MR images acquired using MR scanners of different vendors and characterised by different magnetic field intensities (1.5 and 3 T).

## Methods

### Patients

A total of 195 LARC patients treated in two different medical centres (XXX, and YYY) were enrolled.

The cohort coming from Institution A (136 cases) was retrospectively enrolled among patients treated between May 2008 and December 2014; the cohort coming from Institution B (59 cases) considered patients among those treated between November 2008 and March 2012.

The inclusion criteria of this study were: patients affected by biopsy proven LARC with no evidence of distant metastases at staging radiological exams (Chest-Abdomen contrast enhanced CT and pelvic MRI); age of 18 years or more at the time of diagnosis.

No differences based on gender, age or ethnic group were considered for patient selection purposes. Informed consent was acquired from all the patients according the two ethical committees’ policies.

### Treatment workflow and response assessment

All the patients were treated with to the same protocol: neoadjuvant long course chemo-radiotherapy followed by total mesorectal excision 6–8 weeks after the end of nCRT. The 25 fractions radiotherapy treatment was administered using a simultaneous integrated boost technique (55 Gy in fractions of 2.2 Gy to Gross Tumour Volume (GTV) and corresponding mesorectum and 45 Gy in fractions of 1.8 Gy to whole mesorectum and selected lymphatic drainage stations, according to disease stage) or a technique without a boost (50 Gy in fractions of 2.0 Gy to GTV, mesorectum and elective lymph node stations). Concomitant chemotherapy with chronomodulate Capecitabine (1650 mg/mq) or intravenous 5-Fluorouracil (5-FU) or an intensified schedule with Capecitabine (1300 mg/mq) plus Oxaliplatin (60 mg/mq) was prescribed, depending on the clinical stage and general conditions of the patient.

The pathological reports included histology, grading and tumour regression grade (TRG) according to Mandard classification [25]. Pathological complete response was defined as ypT0N0 or ypT0/ypNx.

### Image analysis

The MR images were acquired using a protocol with T2-weighted images acquired in the plane orthogonal to the tumour longitudinal axis. The data were acquired using a 1.5 T MR scanner (GE Signa Exite, Little Chalfont, United Kingdom) in institution A, and a 3 T MR scanner (Philips Medical System, Eindhoven, The Netherlands) in institution B. No intravenous contrast agents were administered for both cohorts of patients.

The images had a spatial resolution of 0.703 × 0.703 mm^2^ for 1.5 T and 0.45 × 0.45 mm^2^ for 3 T. Slice thickness was 3 mm for 1.5 T and 3.5 mm for 3 T MR images.

The field of view was 18 cm for 1.5 T and 14 cm for 3 T MR images.

MR images were imported in a radiotherapy workstation (Eclipse, Varian Medical System™, Palo Alto, California, US) and the GTV was contoured by two experienced radiation oncologists, using the ICRU n.83 guidelines [26].

The DICOM files were imported in Moddicom, an R package (R Core Team, Vienna, Austria) designed for radiomics analyses of biomedical images [27, 28]. All the images were resampled to a spatial planar resolution of 0.7 × 0.7 mm^2^ prior to their quantitative analysis.

### Feature extraction

A total of 90 radiomic features belonging to four families (fractal, statistical, textural and morphological features) were extracted from the images. Two image filters have been applied to the MR image before the feature extraction.

The intensity-based (IB) image filter proposed by Cusumano et al. was applied to the MR images before to extract radiomics features [22]. This approach consisted of normalising the signal intensity of the pixels inside the region of interest (ROI) analysed using as extremes the first and 99-th percentile of the grey-levels histogram representing the ROI. Pixel clusters were then identified considering two threshold levels (lower and upper level) defined as percentages of the maximum intensity level. All the images were filtered considered the IB filter with all the possible combinations of thresholds “lower level–upper level” for levels ranging from 0 to 100% by 10% (55 steps).

Considering the application of all the filters used, a total of 496 parameters were assessed.

### Data analysis

A database was created combining the image features with clinical parameters (sex, age, clinical TNM staging) and outcome data (complete or not pathological response). Before merging the two cohorts of patients to a single training set, the homogeneity between the two datasets in terms of clinical parameters and pCR rates was assessed. The homogeneity was estimated using the Wilcoxon–Mann–Whitney (WMW) and Pearson’s *χ*^2^ test, depending on the nature of the variable investigated (WMW for continue variables, *χ*^2^ for categorical ones) [29].

Features were standardised with *Z*-score normalisation before features selection. Both cohorts were considered as training set for data analysis purposes, using cross-validation methods to test the model [29, 30].

Features univariately associated to response (pCR vs no pCR) were selected using WMW tests or *t* test, depending on the normality of data distribution, previously assessed using Shapiro–Wilk test [16, 22].

These tests were separately applied for the two cohorts of patients, and correction for multiple comparisons was performed by using the Benjamini–Hochberg method [18].

The correlation between significant features was evaluated in terms of Pearson Correlation Coefficient (PCC) [30].

The significant features were sorted in function of the *p*-value obtained at the univariate analysis. The feature showing the lowest *p*-value in the 1.5 T dataset was combined with the most significant one obtained at 3 T, and their combination was considered as starting point of the multivariate analysis.

Multiple logistic regression models were created adding as third feature one selected among those resulting significant at least in one of the two datasets.

The models were trained on the whole dataset, obtained merging 1.5 T and 3 T patients, and evaluated in terms of AUC and Aikake Information Criteria (AIC) [31]. The robustness of the predictive model was then evaluated using a tenfold cross-validation analysis with 300 repetitions as cross-validation method.

**Fig. 1:**
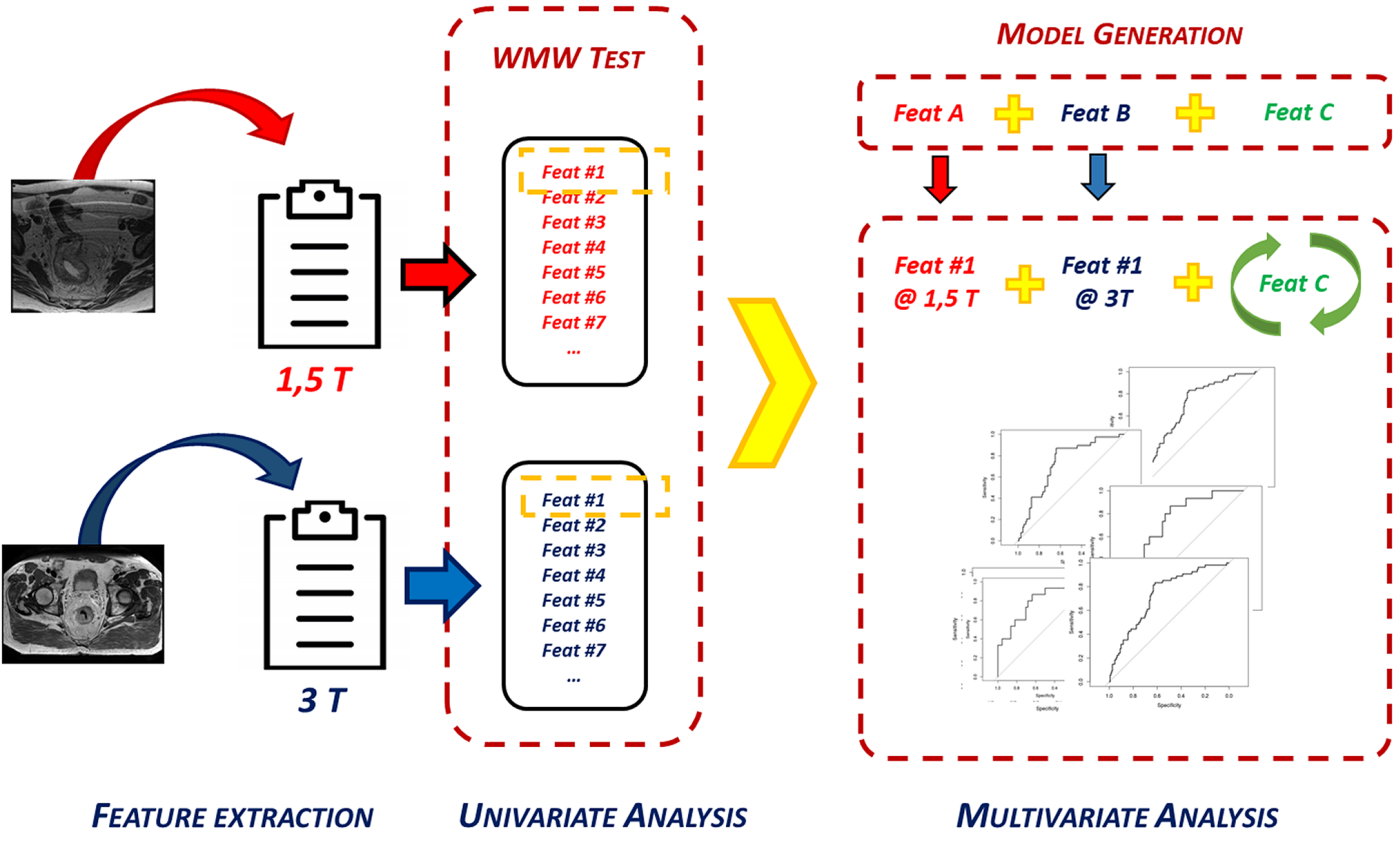
Schematic representation of the workflow adopted for data analysis: feature A was the most significant feature at 1.5 T; feature B was the most significant feature at 3 T; feature C was the feature that combined with feature A and B allowed to generate the predictive model with the highest AUC value

Finally, the model was also applied to the two separate datasets to evaluate its performance when used on MR images acquired at the same magnetic field intensity. In Fig. 1, the adopted workflow for feature selection is graphically depicted.

## Results

The clinical characteristics of the patients enrolled in the study are reported in Table 1. The clinical tumour staging at the time of diagnosis was reported according to the TNM AJCC-UICC classification [32].

**Table 1:** Patients characteristics and descriptive statistics of variables in the 1.5 T and 3 T cohorts

Patient characteristics	1.5 T cohort		3 T pts cohort		*p*-values of differences	
	Mean	Range	Mean	Range	*χ*^2^ test	MW test
Age (sex)						
M	64.0	28–84	63.0	40–79	–	0.57
F	61.9	43–80	64.0	49–76	–	0.69
Age (overall)	63	28–84	63	40–79		0.87
Clinical characteristics						
*N*	%	*N*	%	*χ*^2^ test	MW test	
Response						
pCR	30	22	15	25	0.74	–
not pCR	106	78	44	75		
cT						
3	89	65	49	83	0.13	–
4	47	35	10	17		
cN						
0	6	4	2	3	0.44	–
1	43	32	5	8		
2	87	64	52	88		
Stage						
IIA	6	4	2	3	0.25	–
IIB	0	0	0	0		
IIIB	97	72	44	74		
IIIC	33	24	13	22		

The Pearson’s *χ*^2^ test (*χ*^2^ test) for categorical variables and the Mann–Whitney test (MW test) for continuous ones were used as statistical tests

No statistically significant difference was observed between the two cohorts of patients. A total of 30 patients showed pCR (TRG = 1) in institution A, with a pCR rate equal to 22%. A similar pCR rate was obtained in institution B (25%) where 15 of 59 cases were complete responders.

Table 2 reports the five features showing the highest significance at the univariate analysis in the cohort of the patients acquired using a 1.5 T MR scanner.

**Table 2:** Values of significance in terms of p-value obtained at the univariate analysis for the cohort of patients acquired using a 1.5 T magnetic resonance scanner

Feature (1.5 T Cohort)	Filter	*p* value
Max fractal dimension	Intensity based (40–80)	9.308 × 10^−3^
Median fractal dimension	Intensity based (0–50)	9.744 × 10^−3^
Skewness	Laplacian of Gaussian (0.7 mm)	1.283 × 10^−3^
Variance	Intensity based (10–30)	3.623 × 10^−3^

Table 3 reports the same values obtained for patients acquired with 3 T MR scanner.

**Table 3:** Values of significance in terms of *p*-value obtained at the univariate analysis for the cohort of patients acquired using a 3 T magnetic resonance scanner

Feature (3 T cohort)	Filter	*p* value
Energy	No	9.028 × 10^−7^
Run length non-uniformity	Laplacian of Gaussian (0.35 mm)	2.951 × 10^−5^
Asphericity	Intensity based (10–20)	4.296 × 10^−5^
Uniformity	Laplacian of Gaussian (0.7 mm)	5.163 × 10^−5^
Compactness	Intensity based (10–30)	7.393 × 10^−5^

The features and the relative coefficients characterising the statistical model able to predict pCR are reported in Table 4.

**Table 4:** Parameters and relative coefficients of the proposed predictive model

Feature	Coefficient	SD	*p* value
Intercept	− 1.590	0.218	< 0.001
Max fractal dimension (intensity based: 0–50)	− 0.507	0.212	0.005
Grey-level non-uniformity of run length matrix (intensity based: 0–50)	− 0.621	0.264	0.013
Energy	− 1.393	0.505	0.015

The first two features selected in the model were the *maximum FD,* calculated on the pixel clusters individuated using the IB filter with 0% as lower level and 50% as upper level (IB: 0–50), and the *energy* calculated on the raw image.

The grey-level non-uniformity (GLNU) calculated on the run length matrix (GLNU_RLM) for the same pixel cluster chosen for maximum FD (IB: 0–50) was chosen as third feature, as it ensures the predictive model with the highest AUC.

The AIC value of the developed model was equal to 192.23, that was the lowest value obtained using 3 features. No additional features were added to the model, as the addition of a fourth feature resulted in increasing the AIC.

**Fig. 2:**
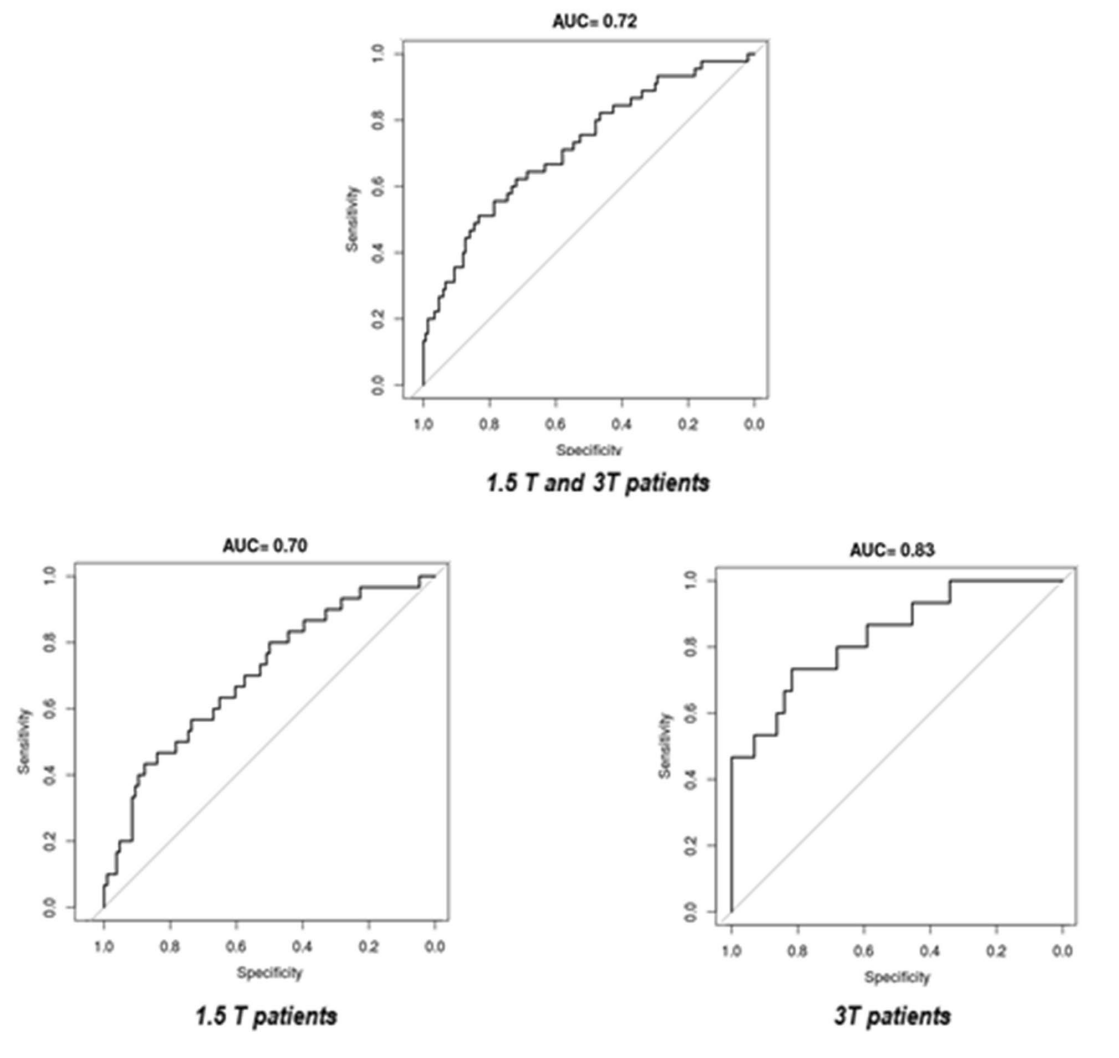
ROC curves: in the upper part of the figure the ROC curve of the merged dataset after cross-validation is depicted. The ROC curves of the 1.5 T and 3 T datasets are shown in the lower part

Figure 2 reports the ROC curves of the elaborated model. The AUC of the model tested on the whole dataset after the cross-validation was 0.72. The resulting AUC for the 1.5 T cohort was 0.70, while 0.83 for 3 T.

## Discussion

In this study, a MR radiomics prediction model for pCR after nCRT in LARC was developed. The pCR rates observed in the two centres (22% in Institution A and 25% in Institution B) were consistent with the clinical data reported in the literature [7, 33].

To our knowledge, this predictive model is the first model able to show promising performances in pCR prediction mixing 1.5 T and 3 T MR images.

This study demonstrates that by using appropriate feature selection methods, it is possible to elaborate predictive models able to overcome the variability due to different magnetic field intensities.

The small p-values in Table 4 demonstrate that all the parameters in the model have discriminating powers. This indicates that with the strategy for feature selection and model elaboration presented in this study it is possible to create predictive models.

Different MR-based prediction models for response to nCRT for LARC patients have been published using images acquired at fixed magnetic field intensity [14, 16, 22, 34]. Intven et al. analysed 3 T MR images, combining features coming from different MR sequences (T2 weighted, diffusion weighted and dynamic contrast enhanced images) and different acquisition timing (before and after CRT) to predict pCR [35]. The authors obtained a very high accuracy (AUC = 0.93 analysing the difference on the ADC maps) but the analysis was done in a relative limited cohort of patients (55 cases) without external validation.

Dinapoli et al. trained a statistical model on an internal cohort of 162 patients and then validated the model on an external dataset of 49 patients from two different institutions. This model, based on the image intensities of T2 weighted MR images acquired before CRT with using 1.5 T MR scanners belonging to different vendors, reports AUC values of 0.73 for the internal and 0.75 for the external dataset [16]. Cusumano et al. added new features coming from fractal analysis to the aforementioned model, aiming to better describe the tumour heterogeneity in the case of LARC patients. The obtained predictive model integrating such fractal features reached AUC of 0.77 ± 0.07 in the internal dataset and 0.79 ± 0.09 in the external one [22]. The AUC of 0.72 obtained in this study combining 3 T and 1.5 T images was comparable to those found in the 1.5 T studies.

The univariate analysis carried out in this work has highlighted the superiority of the 3 T MR images in providing more predictive features respect to 1.5 T MR ones. The most significant feature at 1.5 T shows a *p*-value equal to 10^−4^ (maximum fractal dimension for pixels with intensity lower than 50%), while at 3 T *p*-value is 10^−7^ (energy without applying any image filter). The higher prognostic potentiality of 3 T MR images can be ascribable to the tighter spatial resolution of the images and the higher signal to noise ratio.

One of the limitations of this study is represented by the absence of an independent external dataset composed by MR images acquired at 1.5 T and 3 T in third institution. In absence of these data, the robustness of the model here proposed has been confirmed by the results obtained applying a cross-validation method using a high number of folders and repetitions. A perspective validation of the model proposed will be carried out in the next future, including also data from different centres. However, the results of this study demonstrate that most of the technical aspects that today limit the radiomics diffusion, such as the variability of MR scanners in field strengths and manufacturers, can be overcome by means of dedicated strategy of data analysis.

## Conclusions

In conclusion, a MR radiomics prediction model for pCR after neoadjuvant therapy in locally advanced rectal cancer was developed: the model showed good performance, even when data from patients scanned on 1.5 T and 3 T were merged. This demonstrates that the magnetic field intensity variability can be overcome by means of selecting appropriate images features.

The possibility to overcome the inter-scanner variability and to predict pCR before CRT treatment opens innovative scenarios in cancer care, adding new evidence towards fully personalised approaches and treatment tailoring based on patient-specific tumour heterogeneity description.

## Abbreviations

AIC: Aikake information criteria
AUC: Area under curve
GLNU: Grey-level non-uniformity
IB: Intensity based
LARC: Locally advanced rectal cancer
LE: Local excision
LOG: Laplacian of Gaussian
MRI: Magnetic resonance imaging
nCRT: Neoadjuvant chemo-radiotherapy
pCR: Pathological complete response
ROI: Region of interest
T2-w: T2-weighted
TME: Total mesorectal excision
TRG: Tumour regression grade
W&W: Watch and wait
WMW: Wilcoxon–Mann–Whitney
